# Transcript level and sequence determinants of protein abundance and noise in *Escherichia coli*

**DOI:** 10.1093/nar/gku126

**Published:** 2014-02-06

**Authors:** Joao C. Guimaraes, Miguel Rocha, Adam P. Arkin

**Affiliations:** ^1^Department of Bioengineering, University of California, Berkeley, CA 94720, USA, ^2^California Institute for Quantitative Biosciences, University of California, Berkeley, CA 94720, USA, ^3^Computer Science and Technology Center, School of Engineering, University of Minho, Campus de Gualtar, 4710-057 Braga, Portugal and ^4^Physical Biosciences Division, Lawrence Berkeley National Laboratory, Berkeley, CA 94720, USA

## Abstract

The range over which a protein is expressed, and its cell-to-cell variability, is often thought to be linked to the demand for its activity. Steady-state protein level is determined by multiple mechanisms controlling transcription and translation, many of which are limited by DNA- and RNA-encoded signals that affect initiation, elongation and termination of polymerases and ribosomes. We performed a comprehensive analysis of >100 sequence features to derive a predictive model composed of a minimal non-redundant set of factors explaining 66% of the total variation of protein abundance observed in >800 genes in *Escherichia coli*. The model suggests that protein abundance is primarily determined by the transcript level (53%) and by effectors of translation elongation (12%), whereas only a small fraction of the variation is explained by translational initiation (1%). Our analyses uncover a new sequence determinant, not previously described, affecting translation initiation and suggest that elongation rate is affected by both codon biases and specific amino acid composition. We also show that transcription and translation efficiency may have an effect on expression noise, which is more similar than previously assumed.

## INTRODUCTION

Protein production can be costly in energetic terms for the cell, and, therefore, constitutive expression levels and their regulation are thought to have evolved to meet the trade-off between cost and utility (1–3). While the evolutionary optimality of expression may be contentious (4), the current utility of a protein must be directly related to its activity, which is often difficult to measure *in vivo*. While it is possible to measure protein abundance (PA), which is a better proxy for activity, it is easier to measure mRNA transcript abundances at a genome scale, and thus, it has become common to use transcription as a proxy for PA. Nonetheless, many studies in bacteria demonstrate that transcript abundances are only moderately correlated with PAs (coefficient of determination *R*^2^ ∼ 0.17–0.47) (5–8). Hence, >50% of PA variability across the genome must be explained by posttranscriptional processes that affect translation efficiency and protein degradation, though we will not discuss the latter here (9). Additionally, some of these parameters may as well be intertwined in complex ways, for instance, high overall translation efficiency can lead to a higher density of ribosomes protecting the transcript from degradation, and thereby, affect absolute transcript abundance (10). For these reasons, methods that enable estimation of the individual contribution of the multiple mechanisms to the steady-state level of proteins are necessary prerequisites for understanding the sequence-level trade-offs in expression control and which constrain design of new sequence to meet expression goals (11).

For endogenous genes of prokaryotic organisms, it is generally believed that translation initiation is the rate-limiting step of protein synthesis (12,13). As a result, translation elongation could only be rate limiting by directly impacting initiation rate (13)—for example, by reducing the queue of ribosomes in the mRNA—though the observation that ribosomes in a polysome are well spaced suggests that this may not be the case (14). Moreover, there have been many studies reporting the lack of correlation between the abundance of ribosomes on a particular mRNA and its translation efficiency (15–18). It may also seem more efficient to modulate the expression level of a gene by tuning the efficiency of a promoter and/or the rate of translation initiation, rather than, altering multiple codons of a gene to tweak its translation elongation rate (13). Nonetheless, the nonrandom utilization of the different synonymous codons (i.e. those encoding the same amino acid) is pervasive in nature. The natural selection theory for such codon biases (13) posits that they result from the adaptation to tRNA pools, being more noticeable in highly expressed genes because these are subject to a greater pressure for translation accuracy (19) and efficiency (20). Though there is some evidence that codons are translated faster by more abundant cognate tRNAs (21), large-scale measurements of endogenous mRNA and protein levels have both successfully (22,23) and unsuccessfully (5,11) shown a significant correlation between translation efficiency and codon bias in different organisms. Additionally, a recent study using a synthetic library composed of 154 synonymous genes encoding the same fluorescent protein in *Escherichia coli* showed that the formation of RNA structure inhibiting initiation, rather than the codon bias, was the main determinant of protein synthesis rate (24). The apparent inconsistency between these observations demands for a more thorough scrutiny of both past and recently discovered translation efficiency determinants.

Trade-offs in the mechanisms that affect the steady-state levels of proteins also affect the dynamics of their expression and the heterogeneity of expression over time and across the population. Gene expression is governed by inherently stochastic biochemical reactions that produce the corresponding mRNAs and proteins (25,26). As a consequence, differences in expression can arise within genetically identical cell populations (expression noise) subject to constant environmental cues. In prokaryotes, previous studies have shown that both transcriptional and translational regulation can affect expression noise (27–30), and it has been suggested that translational bursts have the largest effect on cell-to-cell variability (29,30). Conversely, transcriptional bursting is assumed to be the major determinant of gene expression noise in eukaryotes (31–33), although a recent computational study proposes that the effect of translation may be more prominent than previously thought (34).

The availability of large-scale data sets of mRNA and PA provides an important resource with which to dissect the multiple determinants of PA and noise, and to untangle the relative contribution of transcriptional and translational control for the observed phenotypes. Here, we investigate the combined influence of mRNA abundance and >100 transcript sequence features, believed to control translation initiation and elongation efficiency, on protein level of >800 genes in *E. coli*. We developed an integrative statistical model to find a minimal set of sequence features capable of predicting PAs on unseen data [via cross-validation (CV)]. The model, comprising 16 predictors, explains 66% of variation of PA genome-wide. We found that mRNA level is the strongest predictor (53%), as previously shown. However, we found that, in contrast to the arguments above, determinants of translation initiation only explain a small fraction of the total variation of PA (∼1%). We confirmed that RNA structures formed in the initiation region might not be as prominent as previously assumed (23,24), and we report a new feature of the translation initiation complex that may be responsible for the efficient dissociation of this complex and consequent initiation of the elongation step. We also showed that elongation-related features are the major determinants of translation efficiency in *E. coli*. Finally, we used our estimates of transcription and translation efficiency to elucidate their impact on the expression noise.

## MATERIALS AND METHODS

### Data sources

We used the transcript and PAs for 824 genes obtained by RNA-seq and protein fluorescence–fusion measurements, respectively, collected from *E. coli* W3110 grown on M9 media and acquired during exponential phase (35). This data set also provides cell-to-cell variability (expression noise) for each of the measured proteins. We retrieved the corresponding genome from GenBank (http://www.ncbi.nlm.nih.gov/nuccore/NC_007779) and used it to compute sequence-related features impacting gene expression. Aberrant genes containing frameshifts or nonsense start codon were removed from final analysis.

We also evaluated the linear association between the mRNA and PA to find genes with extreme deviation from the expected linear relationship (Supplementary Figure S1). We found that 13 genes may be subject to extreme posttranscriptional regulation (residual variance > 3 standard deviations) and that six of them had complex regulation mechanisms that fall outside the scope of this study (e.g. small RNA inhibition). Five of the remaining seven genes were associated with exceptionally complex transcriptional regulation and two are not well studied. Given the outlier nature of these 13 points, they were removed from the final analysis. However, including the seven genes without strong evidence of specific complex translational regulation did not change our main conclusions (data not shown).

### Sequence features

A total of 107 sequence features were computed from two different regions of the mRNA: the translation initiation region (TIR), which we defined as the region between −25 and +30 with respect to the start codon, and the coding sequence (CDS) defined as the region between the start and stop codon inclusive. Sequence features within these two regions have been shown to influence translation initiation and elongation rates, respectively. Features considered in the TIR influencing translation initiation rate include the multiple characteristics of the hybridization complex between the 3′ end of 16S rRNA and the Shine–Dalgarno (SD) sequence, identity of the start codon, distance between the SD sequence and the start codon and formation of RNA structure (24,36–43) (Supplementary Figure S2 and Supplementary Table S1). In the CDS region, we selected features that are likely to impact translation elongation rate: start/stop codon identity, codon usage, amino acid usage, AT/A content, codon adaptation index (CAI) and protein length (44–48) (Supplementary Table S1).

Simulations of single and hybridized structures of RNA were performed using the UNAfold software (49), and in-house Perl scripts were developed to extract relevant features from the predicted RNA structures. SD sequence motifs for each gene were scored using the Patser software (50) and the respective SD position frequency matrix from *E. coli* (51). Details on the sequence features considered in this study can be found in Supplementary Table S1.

### A predictive model of PA and feature selection

To select a minimal complexity explanatory model of PA built from tens of possible predictors, we used partial least squares (PLS) regression. PLS is a method for relating two data matrices (*X*, a matrix with multiple predictors, and *Y*, a matrix with response variables), by a multiple linear regression model. In our case, the variables in *X* are features, such as mRNA abundance or codon usage, and *Y* is simply the PA. PLS finds a dimensionally reduced projection of *X* (components) that captures most of its variance and has a maximum covariance with a similar projection of the *Y* matrix. This is the method of choice for handling multicollinearity among *X* values and, hence, provides a more robust estimation of regression coefficients than simple multiple linear regression. The following equation shows the linear relationship between the response variable and predictors, where the factor interactions were excluded because of the difficulty in the biological interpretation of these terms and because, when included, they did not significantly improve the model performance (data not shown):

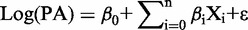



Where x_i_ are the multiple predictors (mRNA concentration and sequence features), β_i_ are the regression coefficients for each of the explanatory variables, β_0_ is the regression constant and ε is the error term. The numeric predictors and response variable were converted to a normalized standard score (z-score) and fitted to the regression model using the package ‘PLS’ (52) for the R software suite (53).

**Figure 1.**
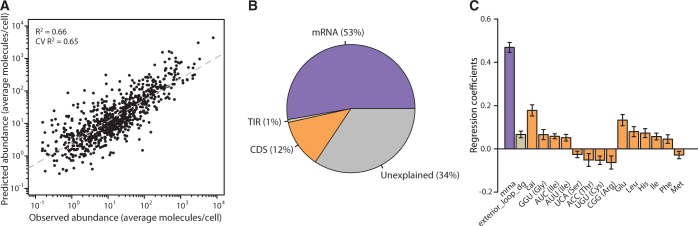
Determinants of PA in *E. coli*. (**A**) Predicted versus experimentally measured protein concentration using a composite model with 16 predictors (R^2^ = 0.66 and CV R^2^ = 0.65). (**B**) Aggregated explanation of PA variation by each group of predictors. (**C**) Regression coefficients for all the predictors in the model. Error bars represent the standard deviation of the regression coefficients based on jackknife variance estimates from 10-fold CV procedure.

We then used stepwise regression with backward selection (54) to down-select the initial 108 predictors to a final set of 16 showing the highest explanatory power (Figure 1A). Specifically, we generated composite models with less complexity by iteratively removing the variables—selected based on the jackknife variance estimates for the regression coefficients—that did not reduce the accuracy of the model, as evaluated by the coefficient of determination of a 10-fold CV procedure.

The performance of the best PLS model was also compared with that of a multiple linear regression model, as well as that of the following nonlinear models: neural networks, support vector machines and random forest. These models were fitted using the algorithms implemented by the package ‘rminer’ (55) for the R software suite. None showed better accuracy than the PLS model (Supplementary Figure S3).

## RESULTS

### Individual predictor performance

Cellular PAs result from the combined effect of multiple mechanisms that tune production and degradation. For example, the steady-state mRNA concentration of a gene is the combined outcome of transcript production and degradation, and, as expected, we identified a strong positive correlation between mRNA and protein levels (Pearson correlation coefficient *r* = 0.7262, *P* ≤ 0.001, Table 1).

**Table 1.** Factors’ individual correlation

Variable	Correlation with PA	Partial correlation with PA given mRNA levels
mRNA level	0.7262***	0
TIR		
16S:SD (exterior loop ΔG)	0.1075**	0.1240***
RBS calculator score	0.1462***	0.1106**
Accessibility	0.0606	0.0912*
Single-stranded bases	0.0630	0.0884*
Folding energy (ΔG)	0.0635	0.0729
CDS		
CAI	0.5828***	0.3526***
ATC	0.3974***	0.2734***
GAA	0.3215***	0.2527***
Ile	0.1933***	0.2319***
Glu	0.2940***	0.2252***

List of the top five predictors with most significant partial Pearson correlation coefficients with PA given the mRNA concentration for each category of features considered. F-test *P*-values were adjusted using false discovery rate (FDR) method (56) to correct for multiple testing: *P ≤ 0.05, **P ≤ 0.01, ***P ≤ 0.001.

We observed that many sequence features are individually moderately correlated with PA and slightly less partially correlated with PA given mRNA levels (Supplementary Figure S4 and Supplementary Table S1). These correlations were still valid when the nonparametric Spearman test was used (data not shown). Bulmer and others have suggested that initiation is the rate-limiting step of translation (12,13). However, our results showed that sequence features related to initiation are generally less correlated with translation efficiency (i.e. PA given mRNA levels) than elongation ones (Table 1). A number of seminal (39,57) and recent studies (24,41) have focused on the propensity of RNA structures to control translation initiation. As previously reported (23), we do not find a significant correlation between folding energy and translation efficiency (*r* = 0.0729, *P* = 0.063). However, we found significant correlations between other features related to RNA structure within the initiation region and translation efficiency (Table 1). As expected, our results suggest that weaker RNA structures in this region contribute to increased protein production (24,39,57). We also observed that a recently developed calculator of translation initiation rates (41), which consolidates several determinants of initiation such as RNA structure and SD sequence strength, is modestly correlated with PA given mRNA levels (*r* = 0.1106, *P* = 0.004). Surprisingly, we found that the binding free energy—lower energy corresponds to tighter binding—of the external loop of 16S:SD hybridization complex is the initiation-related predictor with highest correlation with translation efficiency (*r* = 0.1240, *P* ≤ 0.001), which suggests that weak binding at this particular region can be favorable for translation initiation (Supplementary Figure S2). To further confirm the predictive power of this feature, we used an independent data set composed of many synthetic sequences varying different translation initiation features (41), and found that our predictor was also significantly correlated with PA (*r* = 0.31, *P* = 0.001, *n* = 107).

The influence of codon bias on translation efficiency is a topic of active debate. Several studies advocate that the usage of codons adapted to tRNA may increase protein yields (22,23,58,59), whereas many others failed to find correlations between codon bias and translation efficiency (5,11,14,24,48). Our results show that a genome-wide codon preference metric, CAI, is significantly correlated with PA after controlling for mRNA abundance (*r* = 0.3526, *P* ≤ 0.001). Furthermore, we observed significant correlations for the usage of specific codons (e.g. ATC: *r* = 0.2734, *P* ≤ 0.001 or GAA: *r* = 0.2527, *P* ≤ 0.001) and amino acids (e.g. Ile: *r* = 0.2319, *P* ≤ 0.001 or Glu: *r* = 0.2252, *P* ≤ 0.001). The importance of protein’s amino acid composition has been observed for other prokaryotic (8) as well as eukaryotic organisms (11,22,60,61).

### A composite model to predict PA

We next sought to explore the combined effect of transcription- and translation-related features to predict the steady-state protein concentration across the whole genome. In contrast to previous studies in bacteria, our method yields an integrated model based on a minimal number of explanatory factors that is validated using unseen data (see ‘Materials and Methods’ section). Ultimately, a PLS regression model (Figure 1A and see ‘Materials and Methods’ section) considering only 16 predictors showed the highest accuracy (*r* = 0.81, *R*^2^ = 0.66 and cross-validated (CV) *R*^2^ = 0.65, Figure 1 and Supplementary Figure S5).

Our model integrating mRNA levels and sequence features influencing translation efficiency explained 66% of the variability of PAs experimentally measured for >800 genes (Figure 1A). As expected, transcript abundance was the main determinant of protein concentration (53%), as it encompasses the result of several mechanisms of transcript production and stabilization. CDS features likely controlling translation elongation stand out as the second most important explanatory class (12%), followed by a small, yet significant, contribution of translation initiation determinants (1%) (Figure 1B).

The regression coefficients of the linear model estimate the weight of each predictor on the steady-state concentration of proteins (Figure 1C). The contribution of mRNA level stands out as the dominant effect, immediately followed by the CAI score. The only feature selected influencing translation initiation is the free energy of the exterior loop of the 16S:SD hybridization complex (exterior_loop_dg). Its positive regression coefficient indicates that weak binding in this region is beneficial for translation efficiency. Surprisingly, in addition to CAI, which measures the overall codon adaptation of the gene, we found a set of specific codons and amino acid preferences that further influence PA, presumably by controlling gene’s elongation rate. Because CAI score is defined by the codon composition of a set of highly expressed genes, the usage of this predictor could bias the selection of codon and amino acid preferences. To test that, we built a new model by replacing the CAI score by the tRNA adaptation index (tAI) score, which is an unbiased estimate of codon usage based on tRNA copy numbers. We confirmed that predictors previously selected were also significant in the new model, which indicates that using CAI instead of tAI did not bias our feature selection procedure (data not shown). Therefore, we decided to keep the model with the CAI score instead of the tAI score because the former presented slightly better performance.

Our model suggests that codons GGU (Gly), AUC and AUU (Ile) have a positive effect, whereas UCA (Ser), ACC (Thr), UGU (Cys) and CGG (Arg) seem to be detrimental. These weights are in agreement with both the measured abundance of the corresponding tRNAs (62) and codon usage preferences in *E. coli* (20). Specifically, we observed that codons with a negative regression coefficient are translated by rare tRNAs, whereas the codons translated by abundant tRNAs show a positive regression weight. Lastly, the prevalence of certain amino acids in the protein’s CDS can also enhance (Glu, Leu, His, Ile and Phe) or reduce (Met) PA. Such contributions for Glu, Leu, Ile and Met were expected from early observations by Yamao *et al.* that amino acid usage correlates well with the concentration of the respective tRNAs (63). Perhaps, more puzzling are the positive contributions resulting from the usage of amino acids His and Phe because these are infrequent in endogenous genes and present a high biosynthesis cost to the cell (61).

### Expression profile of *E. coli* genes

**Figure 2.**
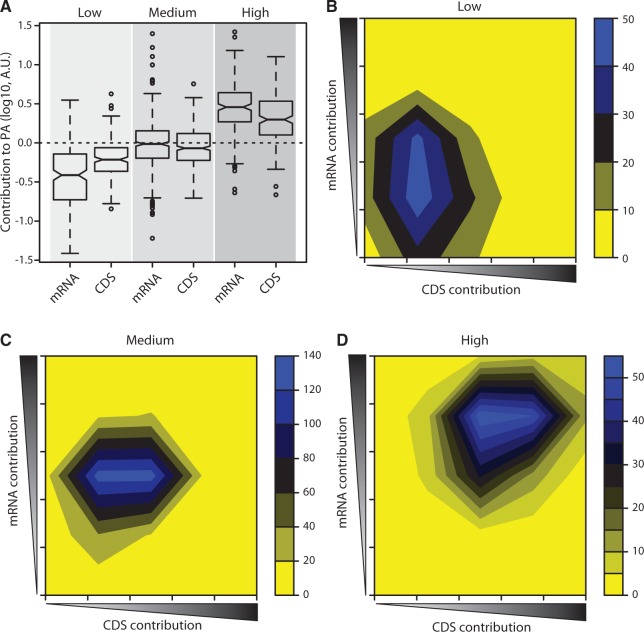
Transcription and translation efficiency act in a concerted fashion. (**A**) Individual contribution of mRNA and CDS features for low (<4 molecules per cell in average), medium and highly (>54 molecules per cell in average) expressed genes. We observed a concerted contribution of mRNA levels and CDS features to the steady-state PA. (**B**) Most low abundant genes tend to be expressed using medium to low levels of mRNA and a low contribution of the CDS features. (**C**) Genes expressed at medium abundance show a balance between mRNA and CDS contribution, where both factors appear most of the time at average levels. (**D**) Highly abundant genes demand for high levels of mRNA and a medium-high contribution of CDS features. Heatmap shade indicates the number of genes.

The developed model integrates a set of predictors that can explain how protein concentration can be tuned at three different levels: transcription, translation initiation and elongation. Hence, we used the model to analyze the quantitative contribution of the different groups of predictors to produce distinct patterns of protein expression. For that, we split gene expression levels into three groups: low, medium and highly abundant genes using the lower and upper quartiles. We then calculated the contribution (i.e. the weighted sum of all explanatory variables belonging to each class) of the two main classes of predictors (mRNA and CDS) to the steady-state protein concentration (Figure 2A).

We observed that mRNA concentration has a lesser/greater median contribution than the CDS features to the low/high abundant proteins. Further, the dynamic range of expression achieved by altering mRNA levels is slightly larger than by altering CDS features. These two main determinants show a concerted effect to achieve the desired protein concentration (Figure 2B–D). For example, the expression of low abundance genes seems to be preferentially attained by expressing mRNA to low/medium levels and by CDS features that tend to correlate with lower expression, whereas highly abundant genes require both high transcription and features encoding efficient translation elongation. We found a significant positive correlation between our model’s estimates of transcription and translation efficiency (defined as the aggregated contribution from TIR and CDS sequence features) (*r* = 0.4405, *P* ≤ 0.001). A similar trend was also observed when we grouped genes by functional classes (Supplementary Figure S6), which suggests these may have optimized different sequence properties to tune transcriptional and/or translational efficiencies. The correlation pattern observed arises from the fact that genes transcribed in greater abundance will also need to be rapidly translated to avoid depletion of free ribosomes in the cell. Although this general trend might be expected, we can certainly observe cases in which the same mean expression can be achieved by trading off mRNA production and translational efficiency. However, it is well-known that this has severe consequences for the dynamics of the response and stochastic behavior of the system.

### Control of expression noise through transcriptional and translational regulation

The data set by Taniguchi *et al.* (35) used in our study provides absolute PA with single cell resolution and, therefore, estimates of cell-to-cell variability of expression levels (noise). One measure of noise is defined by the coefficient of variation CoV = 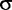/µ, where σ^2^ is the variance and µ is the mean of PA across the cell population. Many single-cell studies have reported a strong dependence between noise and mean expression level (28,31,33,35). However, they have also observed that there can be some deviations from the observed trend (Supplementary Figure S7A). Newman *et al.* (31) defined the difference between the CoV of a particular gene and the median noise expected for proteins with similar abundances to capture this gene-specific expression noise deviation (hereafter noise differential). We calculated the noise differential for all the genes in our data set (Supplementary Figure S7B).

**Figure 3.**
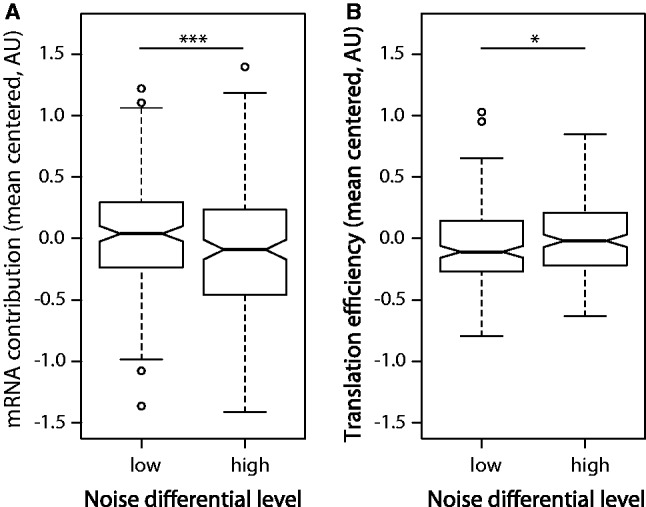
Transcription and translation efficiency affect expression noise. Genes that have noisier expression tend to have less efficient transcription (**A**) and increased translation efficiency (defined as the aggregated contribution of TIR and CDS sequence features) (**B**). The genes were subdivided into two groups: low- and high-noise differential genes, accordingly to the lower and upper quartile of their noise differential levels. High/low-noise differential genes have higher/lower than expected coefficient of variation given the mean expression. Mann–Whitney test significance: *P ≤ 0.05, **P ≤ 0.01, ***P ≤ 0.001.

Many studies have reported the effect of transcription and translation on expression noise, and we used our model’s estimates to evaluate this dependence. For that, we split our gene set into two groups: low- and high-noise differential genes using the lower and upper quartiles. We observed a statistically significant difference in mRNA abundance (Mann–Whitney test *P* ≤ 0.001, Figure 3A) and translation efficiency (Mann–Whitney test *P* = 0.038, Figure 3B) between the two groups with different noise properties. Because we observed a strong correlation between transcription and translation (Figure 2), we further confirmed the significance of the above-mentioned effects by using partial correlation to control for the remaining factor [Spearman rank correlation *rho* (mRNA, noise differential|translation efficiency) = −0.1535, *P* ≤ 0.001; *rho* (translation efficiency, noise differential|mRNA) = 0.1223, *P* ≤ 0.001]. As expected, noisier genes (high noise differential) tend to have lower levels of mRNA and higher translation efficiency than genes with low noise profile. Our results also indicate that transcription and translation contributions to expression noise in prokaryotes may be more similar than previously thought (28–30).

## DISCUSSION

Large-scale transcriptome and proteome measurements provide an invaluable source of information to interrogate the multiple determinants of steady-state protein levels. It is widely accepted that transcript level is the main determinant of protein expression level; however, there is still a significant variation in protein concentration resulting from posttranscriptional regulation. Our results are in good agreement with this view and indicate that 53% of total variation of protein levels can be explained by differential transcript abundances. We also estimated that 13% of the remaining variation could be accounted by factors influencing translation efficiency. Further, it is generally believed that initiation is the rate-limiting step of translation and, as such, codon bias should only have a minor effect on translation efficiency (12,13,24). In equilibrium, the protein synthesis rate will be equal to the number of successful translation elongation termination events per unit time, which will be, at most, equal to the number of ribosomes that initiate translation per unit time. However, a change in the rate of elongation can also lead to enhanced efficiency if it increases the rate of initiation (13) or improves the efficiency of termination. There are two mechanisms by which this may happen: (i) an overall increase in the speed of translation will make ribosomes flow faster and more accurately through the transcript and, hence, become more rapidly available to the pool of free ribosomes (12,13,64)); and (ii) codons translated faster will reduce queuing of ribosomes in the 5′ end of the gene sequence, and lead to more efficient initiation of translation (64–67). Overall, our results suggest a stronger effect of translation elongation- than initiation-related features on the steady-state protein levels, as was previously observed for another bacterium (8) and also for eukaryotes (11,17). However, as described above, the initiation and elongation rates are closely associated and can certainly influence each other. In agreement with this view, a recent whole-cell simulation of translation in *Saccharomyces cerevisiae* thoroughly investigates how the two different steps of translation can affect protein synthesis by tuning ribosome density along mRNAs, as well as the pool of free ribosomes in the cell (64).

Our results clearly indicate a significant impact of codon bias and amino acid usage on translation efficiency (Figure 1B and C), presumably by influencing the elongation rate. We are confident that these effects are directly associated to the adaptation to tRNA pools as confirmed by the regression coefficients estimated from the linear model. For example, the three codons with positive coefficients are recognized by two highly abundant tRNAs, whereas the four negatively weighted codons are recognized by four different tRNAs present in much smaller amounts [∼6 and ∼1.5% of total amount of tRNA in a cell, respectively (62)]. Regression coefficients are also in accordance with codon usage preferences of iso-accepting tRNAs (20) as the slight preference for ATC over ATT can attest (Figure 1C).

Translation initiation rate is influenced by many factors including the affinity between the 16S rRNA and the SD sequence (40), the initiation codon (68) as well as the RNA structure formed in the initiation region (43,57). Genetic alterations perturbing these elements can vary protein synthesis rates up to three orders of magnitude (40,41,57,69). Though we found that many of these determinants are significantly correlated with translation efficiency (Table 1 and Supplementary Table S1), we are surprised to see that our integrative model only selected one initiation-related predictor able to explain ∼1% of the total variation in PA. In agreement with this result, we observed that a recent highly predictive model of translation initiation validated on synthetic sequences (41) is only modestly correlated with translation efficiency of naturally evolved sequences studied here (Table 1). This modest predictive power may be justified by the multitude of translation mechanisms tolerated in *E. coli*, as opposed to more conservative organisms, such as *Bacillus subtilis*, which only recognizes canonical initiation regions (70). Such versatility may hinder the identification of initiation-related determinants by simple models, such as the one used in this study or the ribosome-binding site (RBS) calculator (41), and may also justify the weak SD motif signal observed in *E. coli* endogenous genes (51).

The only translation initiation-related predictor selected by our model (exterior_loop_dg, Supplementary Figure S2) suggests that the free energy of the external loop of the 16S:SD hybridization complex is positively correlated with efficient translation initiation. Though such mechanism has never been identified and, therefore, future experimental evidence is necessary, we speculate that weak binding in this region may have a beneficial effect on expression by facilitating the subsequent disruption of the ribosome from SD sequence to start the elongation stage. Our hypothesis is further supported by a significant correlation found between this predictor and PA of synthetic sequences from an independent data set (41) and by the fact that extremely long complementarity between 16S rRNA and SD sequence does not produce higher translation rates (71).

Lastly, we found a general correlation between our estimates of transcription and translation efficiency, which demonstrate the concerted operation of the two mechanisms (Figure 2). Genes that are transcribed at high rates create an increased demand for ribosomes and, hence, must be efficiently translated to avoid depletion of free ribosomes in the cell and maximize growth (72). Likewise, mRNA transcripts may be more protected from degradation by exo- and endonucleases because of increased ribosome occupancy occluding binding and cleavage sites on the transcript. Transcription and translation are also known to affect expression noise, and previous studies in prokaryotes have suggested that large fluctuations in protein levels result predominantly from low transcription and efficient translation (29,30). Conversely, our analysis shows that both transcription and translation efficiency correlate with expression noise at approximately the same level when controlling for each other. Because tuning transcription or translation efficiency may have similar magnitude but antagonistic effect on expression noise, it allows the independent adjustment of protein average abundance and noise profile for each gene. Interestingly, a recent study in yeast also suggests that the impact of translation on gene expression noise is comparable with that resulting from transcriptional bursting (34), which was previously believed to be more prominent (32,33).

There is still ∼34% of variation of protein levels, which is not explained by our model (Figure 2B) and may result from measurement variability [∼15% as estimated from replicate to replicate variability (35)], as well as other parameters not directly related to the general properties of canonical translation studied here, but to gene-specific regulation (e.g. *trans*-regulation by small RNAs, Supplementary Figure S1). Additionally, protein decay rates have also been shown to impact steady-state protein concentrations (6,11,15,73) and could certainly affect noise.

Our analysis expands the current knowledge by dissecting the contribution of a large number of transcript sequence-related features to differential PA in *E. coli*. In addition to unraveling new determinants with significant impact on translation initiation, we confirm the relevance of codon and amino acid usage to the efficiency of translation. The method developed also quantifies the effect of transcription and translation not only on average protein levels but also on cell-to-cell variability.

Finally, our model can be readily used to predict PAs for all genes of *E. coli* as long as mRNA abundance data sets are available. Additionally, the model was validated on unseen data to ensure a good predictive power. Therefore, it can potentially be used to aid in the computational design of synthetic gene sequence variants tuning the expression levels of both endogenous and heterologous genes in *E. coli*, which can be useful for many applications such as the optimization of metabolic pathways.

## SUPPLEMENTARY DATA

Supplementary Data are available at NAR Online.

## FUNDING

Fundação para a Ciência e Tecnologia [SFRH/BD/47819/2008 to JCG]; the Synthetic Biology Engineering Research Center under National Science Foundation [04-570/0540879]; and the European Regional Development Fund through the COMPETE Programme and by National Funds through the Fundação para a Ciência e a Tecnologia [FCOMP-01-0124-FEDER-015079]. Funding for open access charge: Synthetic Biology Engineering Research Center.

*Conflict of interest statement*. None declared.

## Supplementary Material

Supplementary Data
