# Supplementary material for: Transcript level and sequence determinants of protein abundance and noise in Escherichia coli
Source: Nucleic Acids Res. 2014 Feb 6;42(8):4791–9. doi: 10.1093/nar/gku126 (PMC4005695; doi:10.1093/nar/gku126)
Supplement: Supplementary Data [file supp_42_8_4791__index.html]

Transcript level and sequence determinants of protein abundance and noise in Escherichia coli — Transcript level and sequence determinants of protein abundance and noise in Escherichia coli — Supplementary Data 

# Transcript level and sequence determinants of protein abundance and noise in *Escherichia coli*

## Supplementary Data

files

**Files in this Data Supplement:**

- Supplementary Data - pdf file
