# Supplementary material for: Transcript level and sequence determinants of protein abundance and noise in Escherichia coli
Source: Nucleic Acids Res. 2014 Feb 6;42(8):4791–9. doi: 10.1093/nar/gku126 (PMC4005695; doi:10.1093/nar/gku126)
Supplement: Supplementary Data [file supp_gku126_nar-02905-z-2013-File005.pdf]

SUPPLEMENTARY DATA

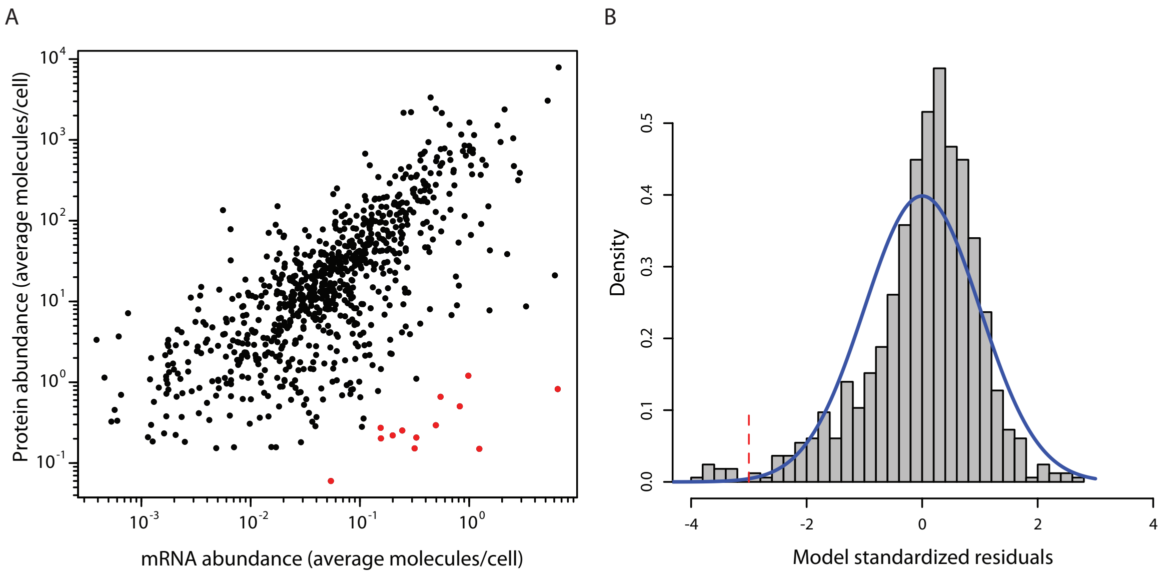

Figure S1. Association between mRNA and protein abundance.

(A) The plot shows a very strong correlation (Pearson correlation coefficient r = 0.7252) between the experimentally measured mRNA and protein abundances, where each point is a gene. Despite the evident association between mRNA and protein abundance, there are a few genes that diverge significantly from the fitted linear regression (highlighted in red). **(B)** Histogram of the standardized residuals for the linear regression between protein and mRNA abundances. The red dashed line indicates the 3σ sigma deviation from the expected mean of the normal distribution of residuals centered at zero (blue line). There were 13 genes with residuals variance greater than 3σ, which are the ones highlighted in red in panel (A). All of them show very high levels of mRNA when compared to the corresponding protein abundances. Among these genes, we found that many of these genes are post-transcriptionally regulated by complex mechanisms. In particular, we found genes that are *cis*-regulated by the formation of long-range mRNA secondary structures (*gnd*) or translation attenuators (*cspG* and *ugpB*) that inhibit translation initiation, as well as other genes (*dppA*, *ompC* and *gltI*) that are *trans*-regulated by small RNAs. Additionally, we found genes that, despite not having any post-transcriptionally regulation described, show a complex transcriptional regulatory architecture composed by many promoters, transcription start sites and transcription factor binding sites (*osmC*, *pykF*, *atpC*, *rbsB* and *rob*), as well as two other genes that were not well studied (*ybgF* and *yiaF*). These observations point out that some of these genes may have very complicated regulatory mechanisms that our model does not intend to cover and hence were removed from the final analyzed dataset.

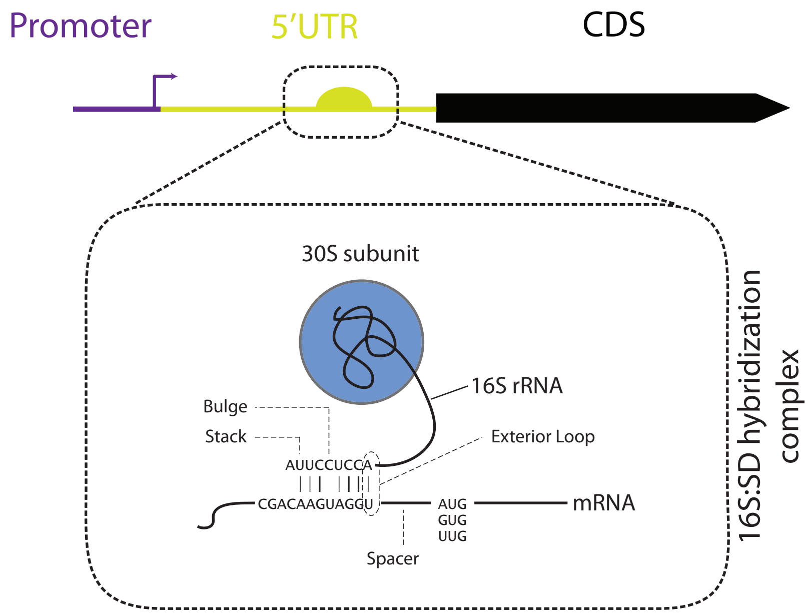

Figure S2. Sequence features of the 16S:SD hybridization complex.

The figure depicts a detailed schematic of the sequence features considered for the 16S:SD hybridization complex. The feature referenced as “Exterior loop” shows a significant positive correlation with protein abundance when controlled for mRNA levels (r = 0.1240, P-value = 0.001). This means that the weaker the binding at the end of the 16S:SD complex, the higher the translation efficiency will be. We hypothesize that this weak binding may facilitate the consequent disruption of the initiation complex to start the elongation step. The double helix RNA structures were predicted using the UNAFold Software and in-house Perl scripts were developed to extract the multiple features from the predicted structure.

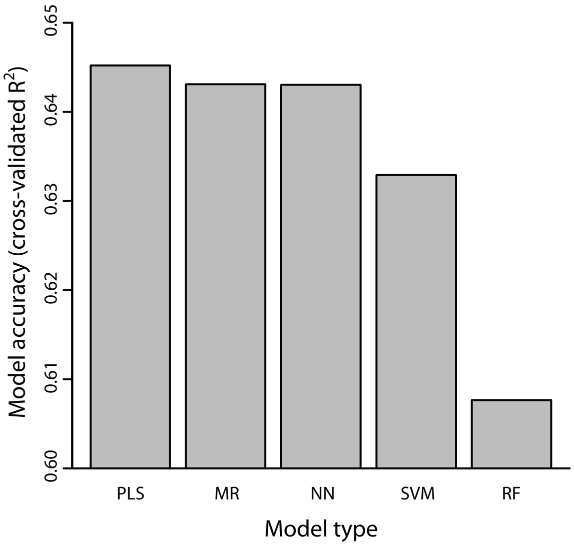

Figure S3. Partial least squares (PLS) regression model shows better accuracy than more complex models.

We compared the accuracy of the PLS model with that of the regular multiple regression (MR), as well as more complex models such as: neural networks (NN), support vector machines (SVM), and random forest (RF). By using the latter models, we expect to encapsulate a more complex behavior of our predictor variables, such as non-linearity and interaction between factors. These models were fitted using the data-mining package *rminer* developed for R. NN and SVM parameters were tuned to yield the best performing model, namely the number of neurons in the hidden layer and the Gaussian kernel parameter, respectively. The RF model was run using default parameters. We show that the PLS model employed in this study has higher accuracy than all the other models. This means that the assumed linear relationship between the predictors and the response variable protein abundance is acceptable and that considering non-linear behavior and interaction between these factors does not yield better results.

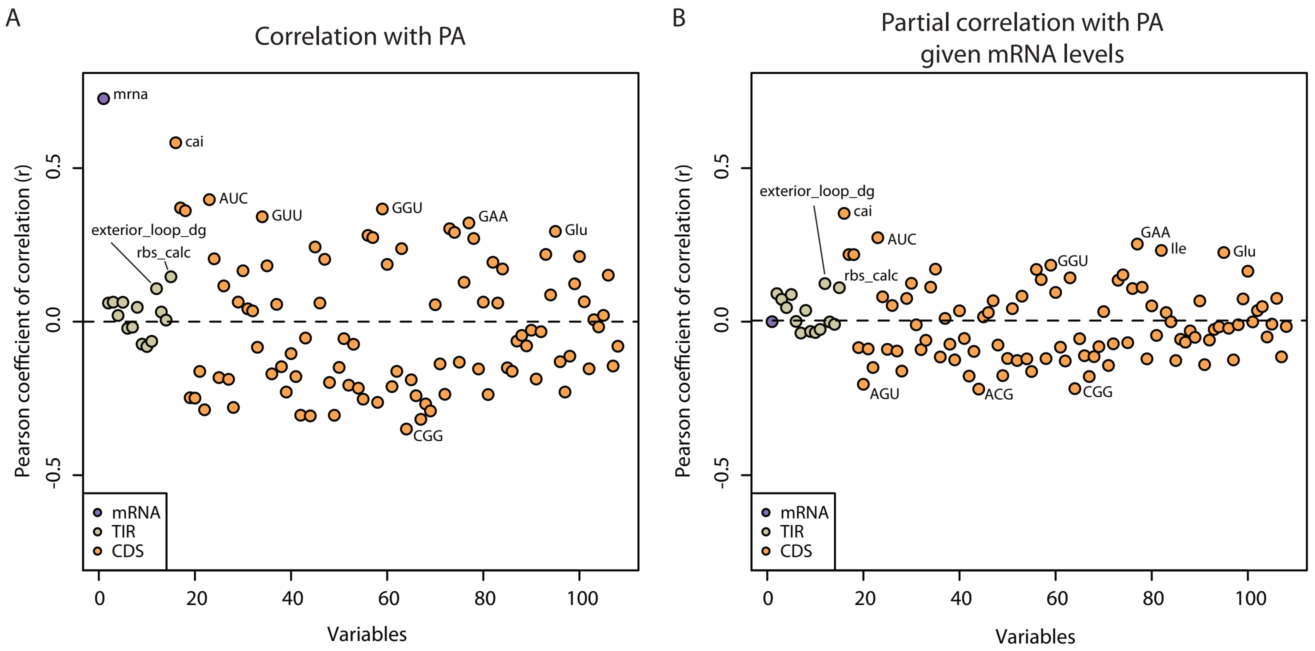

Figure S4. Correlation between features and protein abundance.

The figure depicts the correlation between each of the 108 features considered and protein abundance **(A)**, or protein abundance given the mRNA levels **(B)**. This analysis demonstrates that most of the factors are moderately correlated with absolute protein abundance and slightly less associated with protein levels given mRNA abundances.

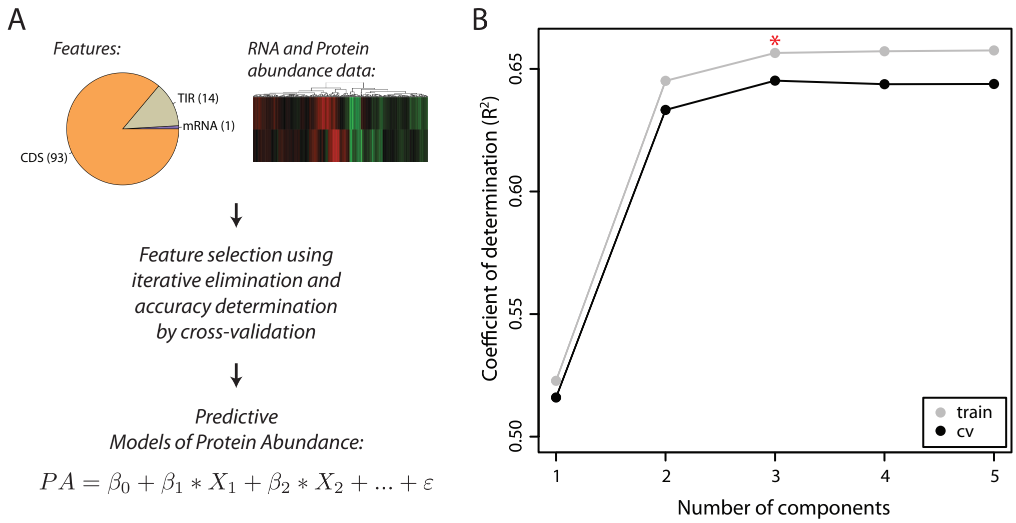

Figure S5. Determinants of protein abundance and composite model search.

(A) More than 100 sequence features were integrated with mRNA levels to predict experimentally measured protein abundances. To select the best composite model, we employed a stepwise regression with backward selection to find the variables with the highest explanatory power (Materials and Methods). **(B)** The plot depicts the PLS model performance — both using the entire training set (gray) and the 10-fold CV procedure (black) — as the number of components increases. A model composed by only 3 principal components (red asterisk), where each component is composed by 10 to 13 features, is enough to capture the maximum covariance between the 16 predictors and the response variable (protein abundance).

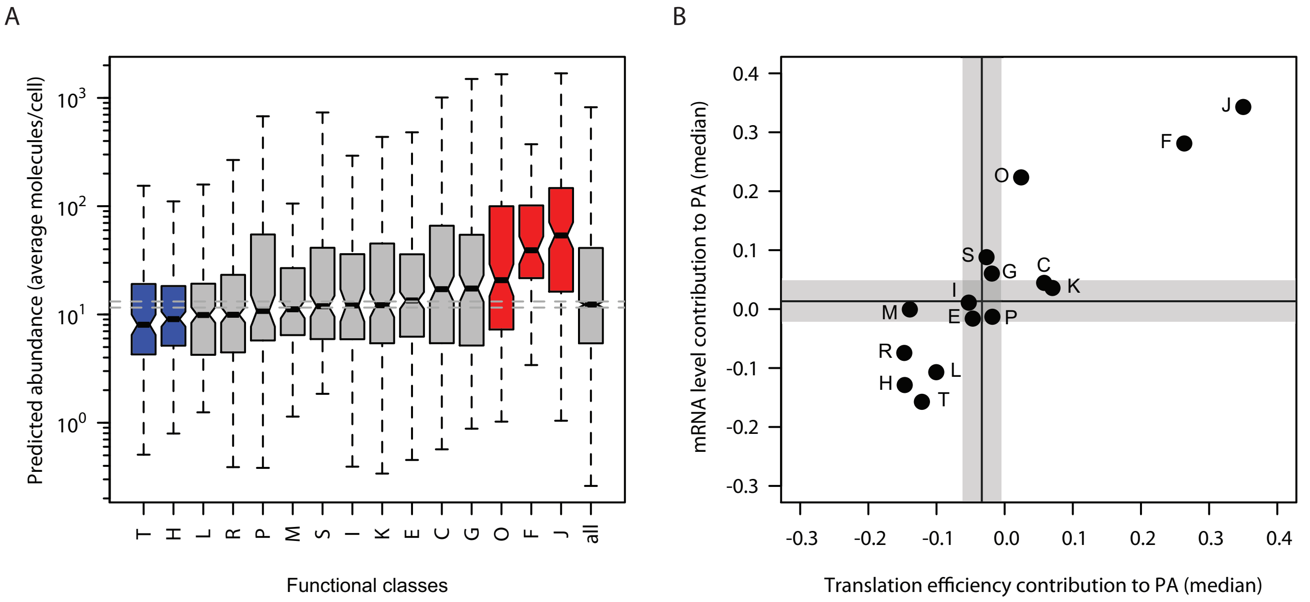

Figure S6. Expression profile by gene function.

(A) Boxplots depict predicted protein concentration by gene function (only functional classes with more than 20 genes were considered). Gene functions showing lower or higher median expression than all proteins are colored in blue and red, respectively. Grey dashed lines indicate the 95% confidence interval around the median abundance of all proteins. **(B)** There is a strong association between the median transcriptional and translational contribution for each functional category (*rho* = 0.81, P-value ≤ 0.001, *n* = 15). Solid lines represent median transcriptional and translational contributions across all proteins and gray shadow indicates the 95% confidence interval of these medians.

Functional groups: T—signal transduction mechanisms; H—coenzyme transport and metabolism; L—replication, recombination and repair; R—general function prediction only; P—inorganic ion transport and metabolism; M—cell wall/membrane/envelope biogenesis; S—function unknown; I—lipid metabolism; K—transcription; E—amino acid transport and metabolism; C—energy production and conservation; G—carbohydrate transport and metabolism; O—posttranslational modifications, protein turnover and chaperones; F—nucleotide transport and metabolism; J—translation, ribosomal structure and biogenesis.

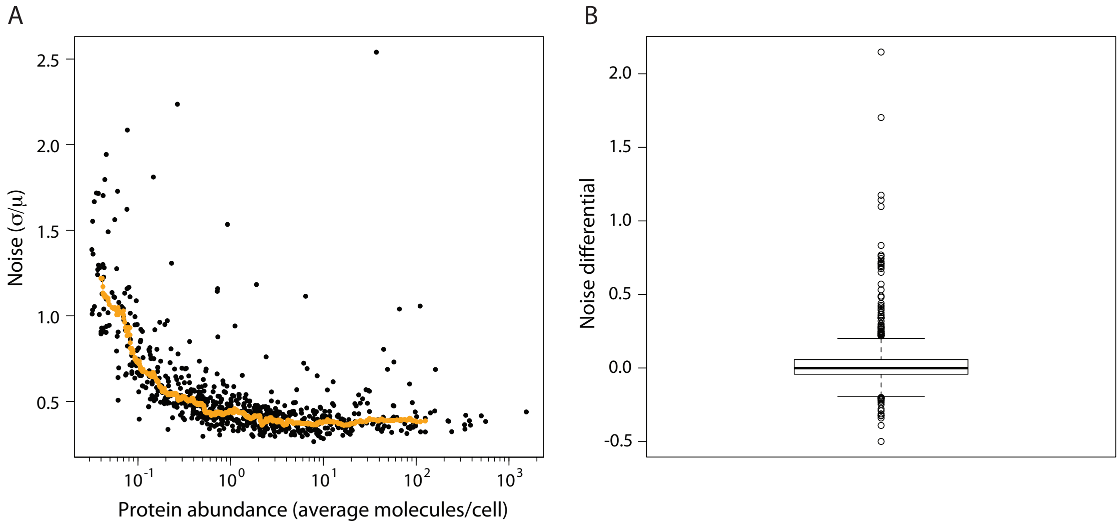

Figure S7. Dependence between noise and mean abundance level, and calculation of noise differential metric.

(A) Noise (as measured by the coefficient of variation) initially decreases quickly with protein abundance and plateaus at higher levels. The median noise across the multiple genes was calculated using a sliding window of 30 data points and is shown in orange. Proteins with very low abundance (less than ~1 molecule per cell) were removed from the analysis due to their extreme noise profile. **(B)** The noise differential is the distance between the noise level of a given gene and the expected median noise level for genes with similar proteins abundance. The boxplot depicts the noise differential for all the genes in our dataset.

Table S1. Factors considered and their correlation with protein abundance.

Pearson correlation coefficients between the factors considered and protein abundance (PA) or PA given mRNA levels. F-test p-values were adjusted using false discovery rate (FDR) method to correct for multiple testing.

Category	Variable	Description	Cor. PA	p.value	Partial Cor. PA given mRNA	p.value
mRNA	mrna	mRNA transcript abundance	0.7262	0.000	0.0000	1.000
TIR	exterior_loop_dg	Binding free energy of the base pair closer to the start codon in the 16S:SD complex	0.1075	0.003	0.1240	0.001
TIR	rbs_calc	RBS calculator score	0.1462	0.000	0.1106	0.004
TIR	accessibility_avg	Average number of single stranded nucleotides in the region [-13,30] with respect to start codon	0.0606	0.101	0.0912	0.020
TIR	single	Number of single bases of the structure in the region [-13,30] with respect to start codon	0.0630	0.090	0.0884	0.023
TIR	fe	Minimum folding energy (MFE) of the structure in the region [-13,30] with respect to start codon (window with highest correlation with PA)	0.0635	0.090	0.0729	0.063
TIR	num_hel	Number of hairpins of the structure in the region [-13,30] with respect to start codon	0.0201	0.590	0.0458	0.257
TIR	spacer	Number of bases between the 16S:SD complex and the start codon	-0.0180	0.626	-0.0371	0.375
TIR	nOfStacks	Number of stacks in the helix formed by the 16S:SD complex	0.0467	0.209	0.0366	0.378
TIR	bulge_mrna	Number of stacks in the helix formed by the 16S:SD complex (mRNA strand)	-0.0810	0.030	-0.0353	0.387
TIR	nOfBulges	Total number of bulges in the helix formed by the 16S:SD complex	-0.0733	0.050	-0.0319	0.438
TIR	bulge_16s	Number of stacks in the helix formed by the 16S:SD complex (16S strand)	-0.0640	0.090	-0.0254	0.535
TIR	sd_spacing	Number of bases between SD motif and the start codon	0.0051	0.884	-0.0087	0.843
TIR	sd_score	Sequence score of SD sequence based on the E. coli SD position weight matrix (PWM)	0.0315	0.400	-0.0009	0.990
TIR	hyb_en	MFE of the helix formed between 16S rRNA and the Shine-Dalgarno (SD) sequence	-0.0218	0.568	0.0008	0.990
CDS	cai	Codon Adaptation Index (CAI)	0.5828	0.000	0.3526	0.000
CDS	ATC	Percentage of occurrences of codon: ATC	0.3974	0.000	0.2734	0.000
CDS	GAA	Percentage of occurrences of codon: GAA	0.3215	0.000	0.2527	0.000
CDS	Ile	Percentage of occurrences of amino acid: Ile	0.1933	0.000	0.2319	0.000
CDS	Glu	Percentage of occurrences of amino acid: Glu	0.2940	0.000	0.2252	0.000
CDS	ACG	Percentage of occurrences of codon: ACG	-0.3069	0.000	-0.2196	0.000
CDS	cu	Codon Usage Bias (based on the bias across all coding sequences)	0.3704	0.000	0.2186	0.000
CDS	cai_ramp	CAI of the first 33 codons (ramp)	0.3613	0.000	0.2184	0.000
CDS	CGG	Percentage of occurrences of codon: CGG	-0.3498	0.000	-0.2182	0.000
CDS	AGT	Percentage of occurrences of codon: AGT	-0.2490	0.000	-0.2039	0.000
CDS	GGT	Percentage of occurrences of codon: GGT	0.3670	0.000	0.1839	0.000
CDS	GGG	Percentage of occurrences of codon: GGG	-0.3181	0.000	-0.1788	0.000
CDS	TTG	Percentage of occurrences of codon: TTG	-0.3047	0.000	-0.1773	0.000
CDS	GGA	Percentage of occurrences of codon: GGA	-0.3048	0.000	-0.1757	0.000
CDS	CAC	Percentage of occurrences of codon: CAC	0.1818	0.000	0.1710	0.000

Category	Variable	Description	Cor. PA	p.value	Partial Cor. PA given mRNA	p.value
CDS	ACT	Percentage of occurrences of codon: ACT	0.2810	0.000	0.1700	0.000
CDS	a_content_init	A content in the region [7,85] (region with highest correlation with PA)	0.2122	0.000	0.1642	0.000
CDS	AAT	Percentage of occurrences of codon: AAT	-0.2525	0.000	-0.1623	0.000
CDS	TCG	Percentage of occurrences of codon: TCG	-0.2794	0.000	-0.1610	0.000
CDS	TTC	Percentage of occurrences of codon: TTC	0.2907	0.000	0.1524	0.000
CDS	CGA	Percentage of occurrences of codon: CGA	-0.2870	0.000	-0.1493	0.000
CDS	CGT	Percentage of occurrences of codon: CGT	0.2378	0.000	0.1427	0.000
CDS	GAT	Percentage of occurrences of codon: GAT	-0.1376	0.000	-0.1427	0.000
CDS	Gln	Percentage of occurrences of amino acid: Gln	-0.1869	0.000	-0.1401	0.000
CDS	GAC	Percentage of occurrences of codon: GAC	0.2740	0.000	0.1370	0.000
CDS	TCT	Percentage of occurrences of codon: TCT	0.3027	0.000	0.1348	0.000
CDS	AGG	Percentage of occurrences of codon: AGG	-0.1618	0.000	-0.1281	0.001
CDS	Ser	Percentage of occurrences of amino acid: Ser	-0.1503	0.000	-0.1268	0.001
CDS	TCA	Percentage of occurrences of codon: TCA	-0.2069	0.000	-0.1265	0.001
CDS	CTG	Percentage of occurrences of codon: CTG	0.1657	0.000	0.1249	0.001
CDS	TGG	Percentage of occurrences of codon: TGG	-0.2292	0.000	-0.1245	0.001
CDS	Trp	Percentage of occurrences of amino acid: Trp	-0.2292	0.000	-0.1245	0.001
CDS	TAT	Percentage of occurrences of codon: TAT	-0.2166	0.000	-0.1211	0.002
CDS	CCT	Percentage of occurrences of codon: CCT	-0.1541	0.000	-0.1210	0.002
CDS	CAA	Percentage of occurrences of codon: CAA	-0.2629	0.000	-0.1202	0.002
CDS	GTC	Percentage of occurrences of codon: GTC	-0.1490	0.000	-0.1198	0.002
CDS	AGA	Percentage of occurrences of codon: AGA	-0.1700	0.000	-0.1150	0.003
CDS	stop.TAG	Identity of stop codon: TAG	-0.1444	0.000	-0.1146	0.003
CDS	CCC	Percentage of occurrences of codon: CCC	-0.2679	0.000	-0.1139	0.003
CDS	GTT	Percentage of occurrences of codon: GTT	0.3416	0.000	0.1123	0.003
CDS	GCT	Percentage of occurrences of codon: GCT	0.2708	0.000	0.1120	0.003
CDS	ATA	Percentage of occurrences of codon: ATA	-0.2412	0.000	-0.1109	0.004
CDS	GGC	Percentage of occurrences of codon: GGC	0.1283	0.000	0.1079	0.005
CDS	CAG	Percentage of occurrences of codon: CAG	-0.0534	0.148	-0.0969	0.013
CDS	ACA	Percentage of occurrences of codon: ACA	-0.1877	0.000	-0.0957	0.014
CDS	TCC	Percentage of occurrences of codon: TCC	0.1866	0.000	0.0952	0.014
CDS	AAG	Percentage of occurrences of codon: AAG	0.0350	0.352	-0.0911	0.020
CDS	AGC	Percentage of occurrences of codon: AGC	-0.1822	0.000	-0.0902	0.021
CDS	TGT	Percentage of occurrences of codon: TGT	-0.1621	0.000	-0.0888	0.023
CDS	GCC	Percentage of occurrences of codon: GCC	-0.2478	0.000	-0.0845	0.031
CDS	TTT	Percentage of occurrences of codon: TTT	-0.2115	0.000	-0.0832	0.034
CDS	ATT	Percentage of occurrences of codon: ATT	-0.0737	0.049	0.0829	0.034
CDS	TTA	Percentage of occurrences of codon: TTA	-0.2908	0.000	-0.0814	0.038
CDS	AAC	Percentage of occurrences of codon: AAC	0.2047	0.000	0.0809	0.038
CDS	CTT	Percentage of occurrences of codon: CTT	-0.1981	0.000	-0.0762	0.054
CDS	stop.TAA	Identity of stop codon: TAA	0.1513	0.000	0.0759	0.054

Category	Variable	Description	Cor. PA	p.value	Partial Cor. PA given mRNA	p.value
CDS	CCG	Percentage of occurrences of codon: CCG	0.0638	0.090	0.0756	0.054
CDS	Gly	Percentage of occurrences of amino acid: Gly	0.1233	0.001	0.0741	0.060
CDS	CCA	Percentage of occurrences of codon: CCA	-0.1467	0.000	-0.0734	0.061
CDS	CTA	Percentage of occurrences of codon: CTA	-0.2366	0.000	-0.0721	0.065
CDS	GCG	Percentage of occurrences of codon: GCG	-0.1320	0.000	-0.0685	0.082
CDS	GTA	Percentage of occurrences of codon: GTA	0.2030	0.000	0.0681	0.083
CDS	His	Percentage of occurrences of amino acid: His	-0.0280	0.456	0.0675	0.085
CDS	Thr	Percentage of occurrences of amino acid: Thr	-0.0633	0.090	-0.0669	0.087
CDS	GTG	Percentage of occurrences of codon: GTG	-0.0835	0.025	-0.0607	0.127
CDS	Asn	Percentage of occurrences of amino acid: Asn	-0.0327	0.385	-0.0600	0.131
CDS	Pro	Percentage of occurrences of amino acid: Pro	-0.1617	0.000	-0.0574	0.151
CDS	CAT	Percentage of occurrences of codon: CAT	-0.1894	0.000	-0.0555	0.167
CDS	CTC	Percentage of occurrences of codon: CTC	-0.1789	0.000	-0.0549	0.170
CDS	TAC	Percentage of occurrences of codon: TAC	0.1164	0.001	0.0526	0.190
CDS	Phe	Percentage of occurrences of amino acid: Phe	0.0637	0.090	0.0514	0.202
CDS	Tyr	Percentage of occurrences of amino acid: Tyr	-0.0789	0.034	-0.0511	0.202
CDS	start.GTG	Identity of start codon: GTG	-0.0172	0.637	-0.0499	0.213
CDS	start.ATG	Identity of start codon: ATG	0.0063	0.865	0.0491	0.219
CDS	Leu	Percentage of occurrences of amino acid: Leu	-0.2374	0.000	-0.0448	0.267
CDS	TGC	Percentage of occurrences of codon: TGC	-0.0553	0.135	0.0423	0.298
CDS	prot_len	Length of protein (in a.a.)	-0.1536	0.000	0.0361	0.382
CDS	CGC	Percentage of occurrences of codon: CGC	-0.1041	0.004	0.0354	0.387
CDS	GAG	Percentage of occurrences of codon: GAG	0.0552	0.135	0.0326	0.429
CDS	Ala	Percentage of occurrences of amino acid: Ala	-0.0448	0.227	-0.0301	0.463
CDS	ATG	Percentage of occurrences of codon: ATG	0.0606	0.101	0.0297	0.463
CDS	Met	Percentage of occurrences of amino acid: Met	0.0606	0.101	0.0297	0.463
CDS	Lys	Percentage of occurrences of amino acid: Lys	0.2190	0.000	-0.0254	0.535
CDS	Cys	Percentage of occurrences of amino acid: Cys	-0.1303	0.000	-0.0214	0.610
CDS	Asp	Percentage of occurrences of amino acid: Asp	0.0871	0.019	-0.0170	0.699
CDS	stop.TGA	Identity of stop codon: TGA	-0.0800	0.032	-0.0157	0.717
CDS	AAA	Percentage of occurrences of codon: AAA	0.2432	0.000	0.0156	0.717
CDS	ACC	Percentage of occurrences of codon: ACC	0.0563	0.129	0.0105	0.820
CDS	GCA	Percentage of occurrences of codon: GCA	0.0418	0.261	-0.0102	0.820
CDS	Arg	Percentage of occurrences of amino acid: Arg	-0.1124	0.002	-0.0101	0.820
CDS	start.TTG	Identity of start codon: TTG	0.0204	0.589	-0.0081	0.850
CDS	at_content	AT content in the region [7,85] (region with highest correlation with PA)	0.0648	0.087	-0.0008	0.990
CDS	Val	Percentage of occurrences of amino acid: Val	0.1716	0.000	-0.0005	0.990
